# Prise en charge péri opératoire des urgences chirurgicales abdominales chez l’adulte au CHU Aristide Le Dantec

**DOI:** 10.11604/pamj.2016.24.190.9929

**Published:** 2016-07-01

**Authors:** Ibrahima Gaye, Pape Alassane Leye, Mamadou Mour Traoré, Pape Ibrahima Ndiaye, El Hadji Boubacar Ba, Mamadou Diawo Bah, Mouhamed Lamine Fall, Elisabeth Diouf

**Affiliations:** 1 Service d’Anesthésie-Réanimation CHU Le Dantec, Faculté de Médecine UCAD, Dakar, Sénégal; 2 Service d’Anesthésie-Réanimation HEAR de Fann, Faculté de Médecine UCAD, Dakar, Sénégal

**Keywords:** Anesthésie, périopératoire, urgence chirurgicale abdominale, adulte, Anesthesia, perioperative, emergency abdominal surgery, adult

## Abstract

La prise en charge périopératoire des urgences chirurgicales abdominales reste une préoccupation majeure des anesthésistes du fait des désordres hémodynamiques et/ou métaboliques souvent présents en préopératoire; mais également des complications postopératoires auxquelles elles sont exposées. Les objectifs de ce travail étaient d'étudier les aspects épidémiologiques, diagnostiques, thérapeutiques et pronostiques des urgences abdominales. Etude rétrospective descriptive sur une période de 6 mois portant sur les patients âgés de plus de 16 ans opérés d'une urgence abdominale à l'hôpital Aristide Le Dantec. Les paramètres étudiés portaient sur les aspects épidémiologiques, diagnostiques, thérapeutiques et pronostiques des urgences chirurgicales abdominales. Nous avions colligé 161 cas, près de 20% de l'activité du service. L'âge moyen était de 41 ans [16, 80 ans]. Le sex ratio était de 2, 9. Le délai moyen de consultation était de 4,6 jours. Les péritonites étaient les pathologies les plus fréquentes (25,5%). La fréquence cardiaque moyenne des patients était de 92 bpm (battements/min)et 97 bpm pour ceux ayant eu une préparation hémodynamique préopératoire. La moyenne de la PAM était de 9,66 cmhg et 8,61 cmhg chez les patients préparés. 49,1% des patients étaient de la classe ASA1, 39,9% ASA2, 8,7% ASA3, 2,5% ASA4 et 0,6% ASA5. Une antibioprophylaxie était faite chez 46,30% des patients et 53,41% d'entre eux avaient eu une antibiothérapie.95,6% des patients avaient eu une anesthésie générale et 4,4% une rachianesthésie. La fréquence des incidents peropératoires était de 11,08%. La morbidité était de 4,3% et la mortalité 4,96%. La prise en charge des urgences chirurgicales abdominales doit être multidisciplinaire impliquant anesthésistes, chirurgiens et biologistes afin de réduire davantage le taux de morbimortalité qui reste de nos jours non négligeable.

## Abstract (en)

Perioperative management of emergency abdominal surgery remains a major concern for anesthesiologists due to hemodynamic and/or metabolic disorders often present preoperatively as well as to potential postoperative complications. This study aims to evaluate the epidemiological, diagnostic, therapeutic and prognostic factors of abdominal emergencies. We conducted a retrospective descriptive study involving patients over 16 years old undergoing emergency abdominal surgery at the Aristide Le Dantec University Hospital over a period of six months. The parameters studied were the epidemiological, diagnostic, therapeutic and prognostic factors of emergency abdominal surgery. We collected 161 cases, nearly 20% of the activity in the department. The average age was 41 years [16, 80 years]. The sex ratio was 2.9. The mean consultation time was 4.6 days. Peritonitis was the most frequent pathologies (25.5%). The average heart rate in patients was 92 bpm (beats/ min) and 97 bpm in patients who underwent preoperative hemodynamic preparation. The average Mean Arterial Pressure (MAP) was 96.6 mmHg and 86.1 mmHg in prepared patients. 49.1% of patients were ASA class 1, 39.9% were ASA2, 8.7% ASA3, 2.5% ASA4 and 0.6% ASA5. Antibiotic prophylaxis was used in 46.30% of patients and 53.41% of them underwent antibiotic therapy. 95.6% of patients underwent general anesthesia and 4.4% underwent spinal anesthesia. The frequency of perioperative incidents was 11.08%. Morbidity was 4.3% and mortality was 4.96%. The management of emergency abdominal surgery requires a multidisciplinary approach that involves anesthetists, surgeons and biologists to further reduce morbidity and mortality rate which remains significant even today.

## Introduction

D´après l´OMS, les urgences abdominales chirurgicales sont des douleurs abdominales évoluant depuis quelques heures ou quelques jours (moins de trois) et qui sont en rapport avec une pathologie chirurgicale, nécessitant un traitement en urgence [1]. Elles occupent une place importante dans l'activité des services d'urgences chirurgicales [2]. Leur prise en charge anesthésique reste un exercice délicat pour les médecins anesthésistes réanimateurs, du fait notamment des nombreuses perturbations organiques auxquelles elles sont confrontées. Une étroite coopération interdisciplinaire pendant toute la période périopératoire est d´une grande importance pour le pronostic de ce syndrome potentiellement mortel [3]. Les objectifs de ce travail étaient: d'étudier les aspects épidémiologiques, diagnostiques et thérapeutiques des urgences chirurgicales abdominales de l'adulte au Centre hospitalier universitaire (CHU) Le Dantec; d'évaluer le pronostic des urgences abdominales.

## Méthodes

C'est une étude rétrospective descriptive menée au service des urgences chirurgicales du centre hospitalier universitaire Aristide Le Dantec sur une période de 6 mois (Mars-Août 2014). Ont été inclus tous les patients âgés de plus de 16 ans et opérés pour une urgence abdominale. Tous les patients avaient bénéficié d'une évaluation préopératoire avec un interrogatoire, un examen clinique et des examens complémentaires (biologiques et radiologiques) selon le terrain, la pathologie et son retentissement sur le patient. Au bloc opératoire tous les patients avaient bénéficié d'un monitorage standard: fréquence cardiaque, pression artérielle non invasive, fréquence respiratoire et la saturation périphérique en oxygène. En post opératoire les patients instables ou présentant un retard de réveil étaient transférés en réanimation et ceux stables aux urgences chirurgicales.

## Résultats

Nous avions colligé 161 cas, ce qui représentait 20% de l'activité chirurgicale du service. L'âge moyen des patients était de 41 ans avec des extrêmes de 16 et de 80 ans. Le sexe masculin était prédominant avec un sex-ratio de 2,9. Les pathologies les plus fréquemment rencontrées étaient les péritonites (25,5%), les occlusions (22,9%) et les appendicites (16,8%). L'appendicectomie était le geste chirurgical le plus réalisé avec 43 actes (26,7%). Le délai moyen de consultation était de 4,6 jours [extrêmes 2 hrs-30 jours]. Les signes fonctionnels les plus fréquemment retrouvés étaient: les douleurs abdominales présentes chez 88,2% des patients, les vomissements chez 67,7%, un arrêt des matières et des gaz chez 36%, une diarrhée chez 1,2% des patients. La fréquence cardiaque moyenne des patients était de 92 bat/min [extrêmes 38-160 bat/min]. Les extrêmes de la PAS étaient de 7 et 18 cmHg, la moyenne de la pression artérielle moyenne (PAM) était de 9,66 cmHg.

La numération de la formule sanguine était réalisée chez 147 patients (91,3%), de même que le TP et le TCK. 46 patients présentaient une anémie, 8 patients avaient une thrombopénie et 7 patients avaient un TP inférieur à 50%. Un ionogramme sanguin était fait chez 86 patients (53,41%). Un bilan rénal (urémie et créatinémie) était réalisé chez 85 patients (52,79%). Les examens d'imagerie étaient réalisés chez 72 patients (44,72%): échographie abdominale (54,15%), l'abdomen sans préparation (ASP) (38,87%), scanner abdominal (23,59%). Les patients de la classe ASA1 u étaient au nombre de 79 (49,1%), ceux de la classe ASA2 u 63 (39,1%), 14 patients (8,7%) de la classe ASA3 u, 4 patients (2,5%) de la classe ASA4 u et 1 patient classé ASA5 u (0,6%). Une préparation avec remplissage vasculaire et monitorage en préopératoire était réalisée chez 90 patients (55,9%). La durée moyenne était de 174 minutes [extrêmes 2 hrs-18 hrs]. Les solutés cristalloïdes étaient utilisés dans tous les cas, associés aux colloïdes dans 5% des cas. La fréquence cardiaque moyenne des patients préparés était de 97 bat/min avant cette préparation et de 87 bat/min après. La moyenne de la PAM chez les patients préparés était de 8,61 cmhg avant et 9,13 cmhg après. La diurèse horaire moyenne était de 127 ml [extrêmes 50 - 250 ml]. Une antibioprophylaxie avait été réalisée chez 54 patients (20,5%). L'association amoxicilline-acide clavulanique-métronidazole était utilisée chez 25 patients (46,30%). Une antibiothérapie était réalisée chez 86 patients (53,41%).

Une anesthésie générale avait été réalisée chez 154 patients (95,6%) et 7 patients (4,4%) avaient bénéficié d'une anesthésie locorégionale, à type de rachianesthésie. Une induction à séquence rapide était réalisée dans 96,75% des cas d'anesthésie générale. L'hypnotique le plus utilisé était le thiopental (78% des cas), suivi du propofol (13%) et enfin la kétamine (11%). Le suxaméthonium était utilisé dans 99,32% des cas. Tous les patients opérés sous anesthésie générale avaient reçu du vécuronium. Le fentanyl était utilisé dans tous les cas d'anesthésie générale. Le remplissage peropératoire était fait avec des cristalloïdes dans 95% des cas et avec les colloïdes dans 5% des cas. Six patients avaient bénéficié d'une transfusion, soit 3,73%. La durée moyenne de chirurgie était de 114 minutes [extrêmes de 35 - 330 min]. La durée moyenne de l'anesthésie était de 147 minutes [extrêmes 65 - 380 min]. 19 incidents avaient été notés en per opératoire (11,08%): anesthésiques (11): 10 cas d'instabilité hémodynamique (chute tensionnelle) et une intubation difficile imprévue; chirurgicales (8): 1 brèche colique, 1 issue de selles dans la cavité péritonéale, 1 plaie hépatique, 1 section de la veine fémorale superficielle et 4 brèches iléales

143 patients(92,85%) étaient extubés sur table. Le délai moyen d'extubation était de 18,24 minutes [extrêmes 5 - 40 min]. 11 patients (6,83%) étaient transférés en réanimation en fin d'intervention. Tous les patients avaient reçu des antalgiques par voie intraveineuse en postopératoire. Un patient avait bénéficié d'une analgésie par voie péridurale. 46 patients (27,9%) avaient eu reçu une anticoagulation préventive. Le taux global de morbidité postopératoire était de 4,3%. Il s'agissait d'une péritonite post opératoire dans 71,43% des cas et d'une péritonite persistante dans 28,57% des cas. La mortalité postopératoire était de 4,96% (8 patients) Les causes de mortalité étaient déterminées chez six (6) de nos patients: 2 chocs septiques, 2 chocs cardiogéniques, 1 cas de défaillance multiviscérale et 1 cas d'arrêt cardiaque sur hyperkaliémie sévère.

## Discussion

Les urgences chirurgicales abdominales occupent une partie importante de l'activité des services d'urgences chirurgicales [2]. La population étudiée était jeune (moyenne d'âge de 41 ans) avec une prédominance du sexe masculin (sex ratio 2,9), ce qui est en phase avec les données de la littérature [2, 4]. La péritonite était la première cause d'urgences abdominales chirurgicales avec 25,5% des étiologies. Dans la thèse de Touré [5], les occlusions intestinales arrivaient en tête des étiologies (28% et 34% respectivement). Ceci pourrait être expliqué aux populations plus âgées étudiées par ce dernier (âge moyen (7,3% vs 6,8% de patients âgés de plus de 60 ans) avec une plus grande fréquence des occlusions chez ces derniers. La douleur abdominale était le principal signe fonctionnel des urgences (88,2%) comme rapporté dans la littérature [4, 5]. Le dosage de l'hémoglobine et un bilan de la crase sanguine avaient été réalisés chez 91,3% patients largement supérieurs aux résultats de Touré [5]. Ceci pourrait être expliqué par une prescription presque systématique et non justifiée de la NFS et du bilan de la crase dès l'admission aux urgences chirurgicales. Les examens d'imagerie: l'ASP (17,33%), l'échographie abdominale (24,22%) et la tomodensitométrie abdominale (10,55%) étaient souvent nécessaires pour nous permettre de confirmer le diagnostic suspecté à l'examen clinique. Soumah et al avaient recours à l'échographie et la tomodensitométrie seulement dans 5,68% et 2,27% des cas du fait de l'indisponibilité de ces derniers en urgence. Néanmoins l'ASP était fait à tous ces patients [4].

Dans leur séries Touré [6] et Faboye [6] avaient rapportés des taux de 14,5% et de 15% respectivement de patients préparés (54% dans notre étude), en partie expliqué par un nombre plus petit de patients ASA 1 u. 95,6% des interventions étaient réalisées anesthésie générale alors que dans l'étude de Faboye ce taux était de 51,2% en rapport probablement avec la fréquence des urgences obstétricales dans cette dernière et souvent faites sous rachianesthésie. La séquence hypnotique suxaméthonium puis Sellick à l'induction comme dans les recommandations [7] était fréquemment réalisée (5, notre étude). La fréquence des incidents notés en peropératoire (11,80 %) était de loin inférieure à celles rapportées dans d'autres études africaines [5, 6]. Dans ces dernières séries les chutes tensionnelles fugaces et les accès hypertensifs en per opératoire étaient considérés comme des incidents, ce qui n'était pas le cas dans notre étude. Ce taux élevé d'incidents survenus dans la période peropératoire [5] était sans doute l'explication d'un pourcentage élevé de transfert en réanimation (35,2%) [5]. Il était de 6,83% dans notre étude.

Tous les patients recevaient une analgésie par voie intraveineuse en post opératoire avec du paracétamol, du néfopam et ou du tramadol. Dans la thèse de Touré la morphine était utilisée avec ou sans le paracétamol pour les patients hospitalisés en réanimation. Cependant aucune de ces études n'avaient utilisée la ketamine en post opératoire d'autant plus que son efficacité sur la prise en charge de la douleur post opératoire a été démontrée [8]. La morbidité (4,3%) était sous évaluée dans notre étude car une grande partie des complications n'avaient pas été notées dans le registre. Ailleurs en Afrique la plupart des études montraient des taux très élevés de morbidité [2, 5, 9]. Cette morbidité était en grande partie liée à la survenue de suppurations pariétales en post opératoires, ce qui pose le problème des mesures d'asepsie au bloc opératoire et du bon suivi des plaies opératoires dans les salles d'hospitalisation.

La mortalité globale était de 4,9% avec les états de choc (septique, cardiogénique) comme première cause dans la plupart des études faites en Afrique [9]. La mortalité proportionnelle aux péritonites était la plus élevée, plusieurs facteurs pourraient l'expliquer, notamment le retard de consultation et la mauvaise observance du traitement antibiotique ou encore de leur inefficacité. En ce sens les nouvelles recommandations de la SFAR (Société Française d'Anesthésie Réanimation) pour la prise en charge des infections intra abdominales [10] devraient être plus largement partagées et appliquées dans les différents services de chirurgie abdominale ou mieux encore l'établissement de protocoles d'antibiothérapie adaptés à l'écologie bactérienne de nos services.

## Conclusion

Les urgences chirurgicales abdominales occupent une place importante dans l'activité chirurgicale des services des urgences chirurgicales. Elles nécessitent une prise en charge multidisciplinaire avec une collaboration étroite entre chirurgiens et anesthésiste-réanimateurs. Les causes sont diverses mais les principales restent les péritonites et les occlusions intestinales responsables d'une morbimortalité non négligeable, souvent inhérente au retard diagnostique et à une prise en charge défaillante.

### Etat des connaissances actuelles sur le sujet

- Les urgences chirurgicales sont des pathologies fréquemment rencontrées en milieu chirurgical africain;
- Elles sont grevées d'une morbi-mortalité assez élevée.

### Contribution de notre étude à la connaissance

- La mortalité peut être réduite en instaurant beaucoup plus tôt et de manière rationnelle les antibiotiques.

## References

[1] Vally NT (2013). Fréquence et prise en charge des abdomens aigus chirurgicaux dans le service de chirurgie de l'hôpital provincial de Kananga du 1 janvier 2010 au 31 décembre 2012.

[2] Harouna (2001). Deux ans de chirurgie digestive d'urgence à l'Hôpital National de Niamey: étude analytique et pronostic. Méd Afr Noire.

[3] Freitag B, Schweder R (1993). Acute abdomen-anesthesiologic and intensive care management. Anaesthesiologie und Reanimation.

[4] Soumah SA, Ba PA, Diallo-Owono FK, Toure CT (2011). Les abdomens aigus chirurgicaux en milieu africaIn: étude d'une série de 88 cas à l'hôpital Saint Jean de Dieu de Thiès. Sénégal Bull Med Owendo.

[5] Touré A (2007). Anesthésie pour urgence chirurgicale abdominale à l'hôpital Gabriel Touré de Bamako.

[6] Diop FNM (2013). Prise en charge anesthésique des urgences chirurgicales à l'hôpital régional de Tambacounda.

[7] Debaene B, Lebrun E, Lehuédé MS (1999). Anesthésie pour urgences abdominales. Conférences d'actualisation.

[8] Georges M (2015). Kétamine: hypnotique, analgésique et anti-hyperalgésique. Conférence d'essentiel SFAR.

[9] Rasamoelina N, Rajaobelison T, Ralahy MF (2010). Facteurs de mortalité par les urgences digestives dans le service de réanimation du CHU de Fianarantsoa Madagascar. Rev Afr Anesth Réa Urg.

[10] Montravers P, Dupont H, Leone M, Constantin JM, Mertes PM (2015). Prise en charge des Infections Intra-abdominales. Anesthésie & Réanimation.

